# Impact of a Collaborative Pharmaceutical Care Service for Patients With Parkinson’s Disease

**DOI:** 10.3389/fphar.2021.793361

**Published:** 2022-01-03

**Authors:** Zhan-Miao Yi, Sarah Willis, Yuan Zhang, Na Liu, Qi-Yu Tang, Suo-Di Zhai

**Affiliations:** ^1^ Department of Pharmacy, Peking University Third Hospital, Beijing, China; ^2^ Institute for Drug Evaluation, Peking University Health Science Center, Beijing, China; ^3^ Centre for Pharmacy Workforce Studies, Division of Pharmacy and Optometry, School of Health Sciences, Faculty of Biology, Medicine and Health, The University of Manchester, Manchester, United Kingdom; ^4^ Department of Health Research Methods, Evidence, and Impact, McMaster University, Hamilton, ON, Canada; ^5^ Department of Neurology, Peking University Third Hospital, Beijing, China; ^6^ Clinical Trials Center, National Cancer Center/Cancer Hospital, Chinese Academy of Medical Sciences and Peking Union Medical College, Beijing, China

**Keywords:** Parkinson’s disease, pharmaceutical service, quality of life, drug-related problems, medication adherence

## Abstract

**Objective:** To identify the impact of a collaborative pharmaceutical care service (CPCS) on medication safety and establish the impact of the CPCS on patient reported outcomes for Parkinson’s disease (PD) patients.

**Methods:** Initially, PD outpatients receiving the CPCS between March 2017 and March 2019 were compared with PD patients receiving standard of care to identify differences in management. Pharmacist interventions data were coded and patients with PD receiving the CPCS were compared with those receiving standard of care to determine differences in medicines prescribed and dosage associated with these. Following this, data of patients receiving CPCS at baseline and 3-months follow-up were collected using a questionnaire consisting of validated measures of two patient-reported outcomes [adherence and quality of life (QoL)]. Mean scores for continuous variables were calculated, with descriptive analysis of categorical variables consisting of frequency counts and percentages. Change in adherence score before and after CPCS was investigated using a Wilcoxon sign rank sum test, spearman correlation analysis was used to correlate the changes in QoL before and after CPCS with the number of interventions, and *p* < 0.05 indicates that the difference is statistically significant.

**Results:** A total of 331 PD outpatients received CPCS over 490 outpatient visits with an average age of 71.83 (±12.54). Five hundred and forty-five drug related problems were recorded as pharmacist interventions, of which most involved change to dosage (*n* = 226, 41.47%), adverse drug reactions (*n* = 135, 24.77%), and change in a medication (*n* = 102, 18.72%). Compared with those receiving standard of care, patients receiving CPCS were significantly less likely to have been prescribed pramipexole (18.52 versus 23.77%, *p* < 0.001) and more likely to have been prescribed amantadine (5.40 versus 3.70%, *p* = 0.02) and selegiline (17.36 versus 11.64%, *p* < 0.001). Lower dosages of levodopa/benserazide (0.51 ± 0.31 g versus 0.84 ± 0.37 g, *p* < 0.001), levodopa/carbidopa (0.33 ± 0.23 g versus 0.66 ± 0.47 g, *p* < 0.001), pramipexole (1.14 ± 1.63 mg versus 1.27 ± 0.69 mg, *p* = 0.01), and entacapone (130.00 ± 79.76 mg versus 173.09 ± 97.86 mg, *p* < 0.001) were also recorded. At baseline 119 PD outpatients with an average age of 69.98 (±9.90) were recruited for the longitudinal study. At 3-month follow-up, participants reported improvement in bodily pain subscale (baseline versus 3-months follow-up, 30.04 ± 22.21 versus 23.01 ± 20.98, *p* = 0.037) and medication adherence (6.19 ± 1.50 versus 6.72 ± 1.73, *p* = 0.014). Frequency of CPCS use was related to activity of daily living subscale (*p* = 0.047), the bodily pain subscale (*p* = 0.026), and medication adherence (*p* = 0.011). Total score of PDQ-39 was associated with patient education (*p* = 0.005) and usage and dosage combined with patient education (*p* = 0.006), while medication adherence score was associated with usage and dosage (*p* = 0.005).

**Conclusion:** The CPCS was effective in resolving drug-related problems and in improving patients’ medication regimens, medication adherence, and QoL through patient education and dosage adjustments. This is the first step in the development and feasibility testing of pharmacy services for PD patients in China.

## 1 Introduction

Parkinson’s disease (PD) is a common neurodegenerative disease, with an estimated incidence rate of 37.55 per 100,000 person-years (95% CI 26.20–53.83) in females aged 40 and over and 61.21 (95% CI 43.57–85.99) in males aged 40 years and older, with incidence increasing with age (Hirsch et al., 2016). Patients with PD are likely to be prescribed a large number of medicines to treat motor and non-motor symptoms as well as a number of co-morbid conditions, with levodopa and dopaminergic agonists most commonly prescribed, together with medicines for comorbidities, such as asorthostatic hypotension (McLean et al., 2017). As a consequence of being prescribed multiple medicines, patients with PD face problems with regards to polypharmacy, such as an increased risk of missing doses, and harmful drug related problems (DRPs) associated with drug interactions, as well as reduced quality of life (QoL) and increased treatment burden (Klietz et al., 2019). High levels of polypharmacy associated with complex drug regimens among patients with PD have been linked to poor adherence, reducing therapeutic benefits such as improved mobility, activity, emotional wellbeing, cognition, communication, and body comfort (Daley et al., 2012; Daley et al., 2014). Given this poor adherence to therapy, it is important to understand the causes of this—both to reduce medicines wastage and so that patients get the best possible outcomes from their medicines (Wei et al., 2014).

As a member of the medical team, pharmacists have a key role in ensuring medicines optimisation, especially for patients prescribed multiple medicines and/or those prescribed low therapeutic index drugs (Avery et al., 2012). While to date there is a lack of evidence regarding the contribution of pharmacists to patient outcomes in China, where the role of pharmacists has tended to focus more on medicines supply than on supporting patients to get the best outcomes from their medicines (Yi et al., 2016), studies undertaken in other countries such as Brazil and the United States have demonstrated the contribution of pharmacists to medicines management of patients with PD with patients’ adherence and QoL improved through pharmacists’ interventions (Poon et al., 2012; Foppa et al., 2016). Given this evidence that pharmacists can effectively have a positive clinical impact on patients’ OoL and drug related problems, an innovative service for patients with PD was developed at a 2,024-bed tertiary academic-teaching hospital in Beijing, the collaborative pharmaceutical care service (CPCS).

The CPCS involves a physician and a pharmacist collaborating in providing patient care, with pharmacists working with physicians in the diagnosis and management of patients to provide individualised care for patients. The service is provided in a clinic in the outpatients department. Initially, patients have a consultation with a physician and a pharmacist at the same time. Patients subsequently have a consultation with the pharmacist alone where the pharmacist reviews the patient’s notes to identify drug related problems (DRPs) including potential drug interactions, monitor adverse events (AEs), respond to patient-related medication questions, and provide patient education so that patients get the right medicines and know how to take them correctly, with the intention of improving medicines safety, reducing medicines waste, and of enhancing patient outcomes (quality of life and adherence). During the patient education session, pharmacists provided a leaflet of usage and dosage for patients. Any interventions made by the pharmacist including identification of DRPs are recorded in the pharmacist’s intervention records.

The aim of the study reported here was to investigate the impact of the CPCS. This has been achieved through addressing the following objectives:

1) To identify the impact of the CPCS pharmacist interventions on medication safety by comparing pattern of prescribed medications between CPCS patients and patients receiving standard of care;
2) To establish the impact of the CPCS on patient reported outcomes (adherence and quality of life [QoL]) by comparing the changes seen from baseline to 3-months post-intervention among patients receiving CPCS.

## 2 Materials and Methods

### 2.1 Overview of Study Design

To address the first study objective, records of patients with PD taking part in the CPCS were compared with records of patients with PD receiving standard of care. Patients who received standard of care only visited the physicians without consultation with the pharmacist. For the second study objective, longitudinal analysis of patient reported outcomes (adherence and QoL) captured at baseline (enrolment in the CPCS) and at 3-months follow-up was undertaken to determine impact.

### 2.2 Impact of CPCS on Medication Safety

To identify the impact of the CPCS pharmacist interventions on medication safety (Objective 1) recorded drug related problems (DRPs) and pharmacist interventions were extracted from pharmacist intervention records for the period March 2017 to March 2019. These data were merged with patient demographic information (age, gender, etc.) extracted from outpatient records, with additional data related to patient medication history derived from the hospital electronic prescription information system to create a dataset consisting of patients with PD receiving the CPCS. DRPs and pharmacist interventions data within the dataset were then coded using a validated framework for categorizing pharmaceutical care activities contributing to reducing patient harm associated with detecting DRPs and resolving them (Schaefer, 2002). Severity of AEs was assessed by the Common Terminology Criteria for Adverse Events (CTCAE) v5.0 as Grade 1 to 5 which was widely used in previous studies (Common Terminology Criter, 2017; Smith et al., 2021). Severity of drug-drug interactions was evaluated with Lexi-Interact online (Lexicomp. Drugs interacti, 2021).

Those patients with PD receiving the CPCS were compared with those receiving standard of care to determine differences in medicines prescribed and dosage associated with these. Data related to patients with PD receiving standard of care were obtained from the electronic prescribing database from January 1, 2016, to August 15, 2018.

Data were entered into Microsoft Excel, and the distributions of DRPs and drug use were tabulated. Mean scores for continuous variables were calculated, with descriptive analysis of categorical variables consisting of frequency counts and percentages.

### 2.3 Impact of CPCS on Patient-Reported Outcomes

To establish the impact of the CPCS on adherence and quality of life, data were collected using a questionnaire consisting of validated measures of these patient-reported outcomes. Relevant factors affecting patients’ QoL or medication adherence found in previous studies, such as gender, age, Hoehn-Yahr (H&Y, corrected) grading scale for the evaluation of severity of PD, and other disease related information, were considered at baseline patient characteristics (Schrag et al., 2000; Straka et al., 2018). The H&Y stages 1–2 indicated early stage, H&Y stages 2.5–3 medium stage, and H&Y stages 4–5 advanced stage of PD.

### 2.4 Inclusion Criteria for the CPCS on Patient-Reported Outcomes

Inclusion criteria for the CPCS was as following: (1) patients diagnosed with PD; (2) patients able to communicate; (3) patients able to consent to participate in the study. Patients with dementia or diagnosed with PD symptoms only were therefore not eligible to take part.

Patients with PD receiving the CPCS were followed up 3 months after the baseline measure on the basis of our systematic review on pharmaceutical service for patients with PD expert opinions and the time period used for repeat prescriptions in China.

### 2.5 Patient-Reported Outcomes and Validation

Patients’ adherence was measured using a questionnaire including eight items, in which six items were scored as “yes” = 0 and “no” = 1, another item was “yes” = 1 and “no" = 0; and the last item was by adopting a 5-point Likert scale, namely “never,” “occasionally,” “sometimes,” “frequently,” “always,” corresponding to scores of 1, 0.75, 0.50, 0.25, and 0, respectively. The reliability of the adherence scale was tested with half-reliability test. Change in adherence score before and after CPCS was investigated using a Wilcoxon sign rank sum test, and *p* < 0.05 indicates that the difference is statistically significant.

Patients’ QoL was investigated using the 39 item PDQ-39 scale which consists of eight subscales: mobility (10 items), activity of daily living (6 items), emotional well-being (6 items), stigma (4 items), social support (3 items), cognition (4 items), communication (3 items), and bodily pain (3 items) (Martinez-Martin et al., 2011). For the PDQ-39, a higher score indicates a poorer QoL. Among the influencing factors of patients’ QoL scores, independent variables were used for Mann-Whitney Test *U* test for dichotomous variables; spearman correlation analysis was used for continuous variables; and changes in QoL before and after pharmacological services were measured with Wilcoxon sign rank sum test. Spearman correlation analysis was used to correlate the changes in QoL before and after pharmacological services with the number of interventions, and *p* < 0.05 indicates that the difference is statistically significant.

### 2.6 Sample Size

Sample size was calculated using PASS 11.0 software, with adherence the primary outcome and QoL the secondary outcome. Based on previous literature, where the mean ± standard deviation (SD) of change in adherence score before and after pharmacist intervention was −1.13 ± 0.96 (a lower the adherence score indicates higher adherence) (Zhang et al., 2018), and taking the test level *α* as 0.05 and the power as 0.90, a sample size of *n* = 6 was calculated for the primary outcome. For the secondary outcome, the results of a meta-analysis were used, where findings indicate that the emotional well-being subscale of PDQ-39 before and after the intervention of the pharmacist may have benefits, with a change value of −6.51 ± 24.34 (Mynors et al., 2007). With the test level *α* at 0.05, and the power is 0.80, the sample size here is *n* = 87. In view of the fact that older patients with PD may have a higher drop-out rate, a 30% increase in the sample size was considered and the final sample planned to be included was 113 patients.

## 3 Results

### 3.1 Impact of the CPCS on Medication Safety

#### 3.1.1 Participants’ Demographics

A total of 331 patients with PD with an average age of 71.11 (±10.24 years old) received the CPCS. The service was delivered during 490 outpatient visits; 104 patients had multiple episodes of care. Most were over 65 years-old (*n* = 244, 73.7%) and male (*n* = 181, 54.7%).

**FIGURE 1:**
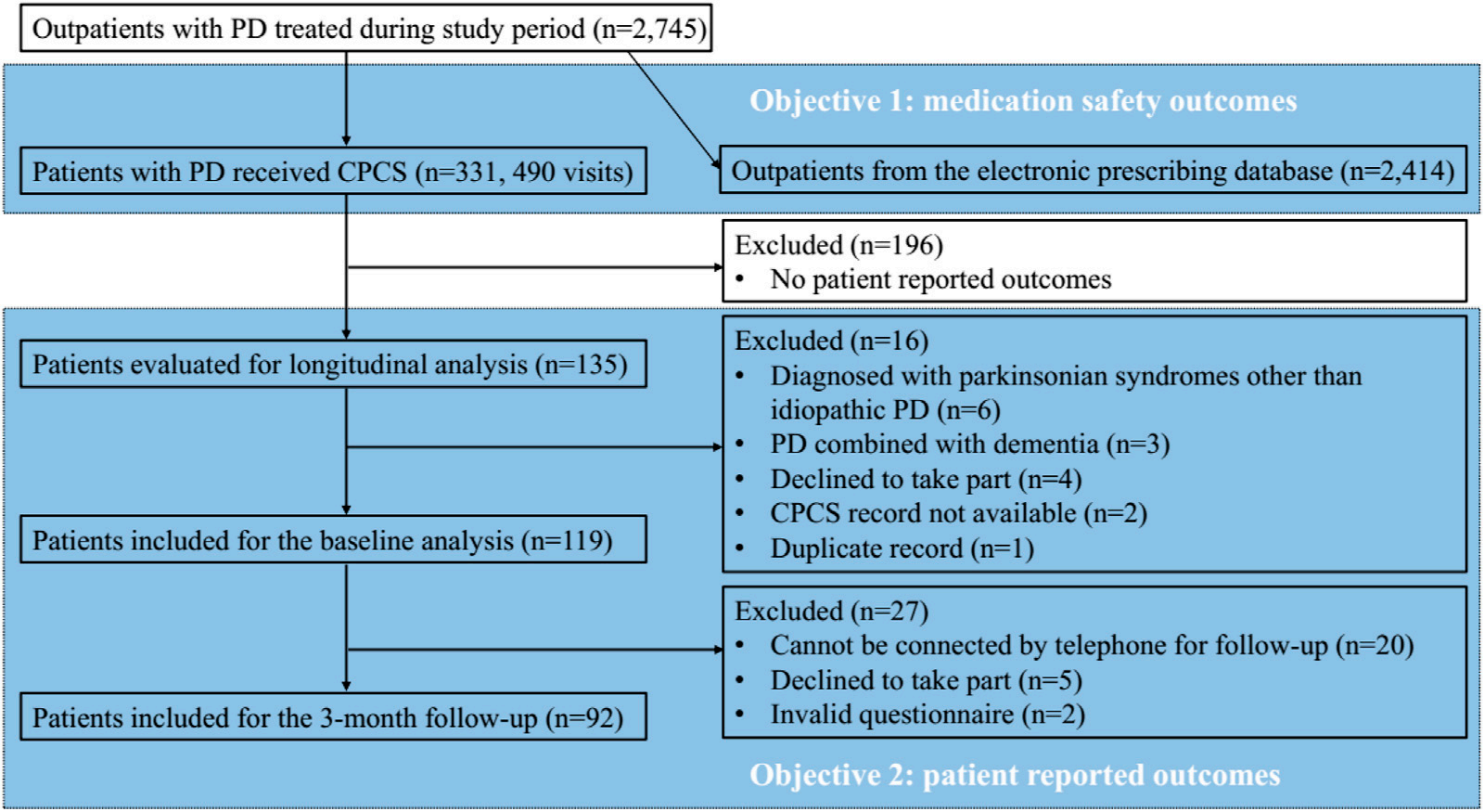
Study participants.

In comparison, 2,414 patients with PD received standard of care. These patients were an average age of 71.83 (±12.54); and as with those receiving CPCS, most were male (*n* = 1,319; 54.64%). There were no statistically significant differences in age (*p* = 0.317) or gender (*p* = 0.988) between those patients receiving the CPCS and those who received standard of care (Figure 1).

### 3.2 Drug Related Problems

A total of 545 DRPs (mean 1.65 per patient) were recorded and interventions made by the pharmacist, of which 226 (41.47%) involved change to dosage, 135 (24.77%) related to AEs, 102 (18.72%) to change in a medication, 51 (9.36%) involved patient education, 18 (3.30%) interventions related to drug-drug interactions, 8 (1.47%) of special complications, and 5 (0.92%) to drug information. Among AEs, 38 (28.1%) were mild AEs (Grade 1) and the rest (71.9%) were moderate AEs (Grade 2). Ninety-nine (73.3%) interventions to AEs were fully accepted and implemented. Among the drug-drug interactions, five (27.8%), two (11.1%), nine (50.0%), and two (11.1%) were classed as “no known interactions,” “no action needed,” “monitor therapy,” and “consider therapy modification,” respectively. All interventions to the drug-drug interactions were fully accepted and implemented.

### 3.3 Prescribed Medicines

Comparing between those receiving the CPCS and receiving standard of care, some difference in medicines were found.

Across all patient age groups, patients receiving the CPCS were significantly less likely to have been prescribed pramipexole [18.52% (223/1,204) versus 23.77% (7,150/30,078), *p* < 0.001] and more likely to have been prescribed amantadine [5.40% (65/1,204) versus 3.70% (1,114/30,078), *p* = 0.02] and selegiline [17.36% (209/1,204) versus 11.64% (3,502/30,078), *p* < 0.001].

Among patients under 65, patients receiving the CPCS were significantly more likely to have been prescribed pirbedil [16.29%, (50/307) versus 11.64% (826/7,096), *p* = 0.014] and selegiline [25.08% (77/307) versus 14.66% (1,040/7,096), *p* < 0.001] and significantly less likely to have been prescribed pramipexole [11.07% (34/307) versus 20.49% (1,454/7,096), *p* < 0.001] and levodopa compound [30.29% (93/307) versus 36.81% (2,612/7,096), *p* = 0.02].

For patients over 65, those receiving the CPCS were significantly less likely to have been prescribed pramipexole than those receiving standard of care [21.07% (189/897) versus 24.78% (5,696/22,982), *p* = 0.011] and more likely to have been prescribed selegiline [14.72% (132/897) versus 10.71% (2,462/22,982), *p* = 0.011].

**FIGURE 2:**
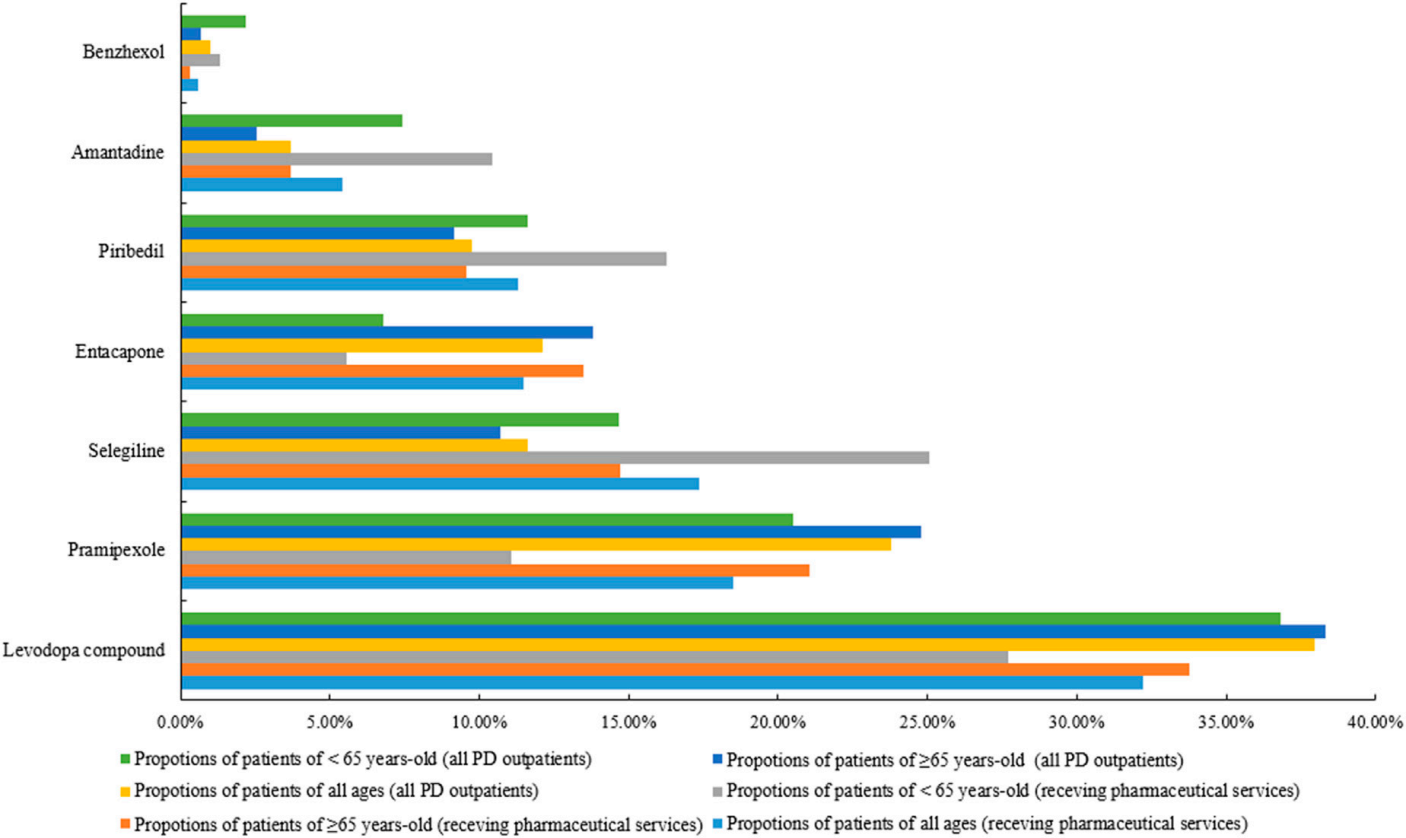
Comparison of prescribing for patients receiving CPCS with patients receiving standard of care.

No statistical significant difference was found in the proportion of prescribed benzhexol and entacapone (Figure 2).

### 3.4 Medicines Dosage

Compared with patients with PD receiving standard of care, those receiving CPCS were prescribed significantly lower dosages of levodopa/benserazide (0.51 ± 0.31 g versus 0.84 ± 0.37 g, *p* < 0.001), levodopa/carbidopa (0.33 ± 0.23 g versus 0.66 ± 0.47 g, *p* < 0.001), pramipexole (1.14 ± 1.63 mg verusu 1.27 ± 0.69 mg, *p* = 0.01), and entacapone (130.00 ± 79.76 mg versus 173.09 ± 97.86 mg, *p* < 0.001).

### 3.5 Impact of the CPCS on Patient Reported Outcomes

#### 3.5.1 Participants’ Demographics

A total of 135 patients were invited to take part in this study. Of these 119 were enrolled, with patients excluded for the following reasons: six were diagnosed with parkinsonian syndromes other than idiopathic PD, three had a diagnosis of PD combined with dementia and hence lacked capacity to consent to participate, 4 declined to take part, two did not have CPCS records, and one duplicated record. The average age of participants was 69.98 years old (±9.90) with a median disease duration of 3.92 years; just over half (53.8%) were male. The median H&Y stage was 2.50, indicating that most participants were in the early stages of PD (see Table 1 for details).

**TABLE 1:** Participant profile at baseline.

Items	Sample size	Category and number
Gender	119	Male/Female: 64/55
Age (year, mean ± SD)	119	69.98 ± 9.90
Course/year (median, interquartile range)	113	3.92 (2.00,6.21)
H&Y scale (median, interquartile range)	119	2.50 (1.50,3.00)
Marital status	109	Married/widowed: 96/13
Education level	119	College and above: 61
		Junior high to high school (including technical secondary school): 51
		Primary and below: 7
Type of jobs	117	Individual, business, enterprise (service staff): 16
		Technology, medical, teachers: 51
		Executive: 17
		Workers: 17
		Farmers/Housewives: 9
		Other: 7
Employment status	118	In-service: 7
		Unemployed: 6
		Retirement: 105
Payment methods	118	Health Insurance: 84
		New Rural Cooperative: 6
		Public expense: 25
		Own expense: 3
Monthly household income	114	<5,000 : 54
		5,000–12,000 : 51
		≥12,000 : 9
Swallowing disorder	119	Yes/No: 23/96
Sleep disorder	119	Yes/No: 61/58
Mental symptoms	107	Yes/No: 45/62
Dysuria	119	Yes/No: 66/53
Constipation	119	Yes/No: 84/34
		Colon cancer: 1
Hyposmia	119	Yes/No: 57/62
Wearing off phenomenon	119	Yes/No: 55/64
On/off phenomenon	119	Yes/No: 22/97

From the 119 participants followed up with consent, 92 took part in the second round of data collection. The reasons for loss to follow-up included: 20 not being available and 5 declined to take part. Among the 92 patients, the average age was 69.69 years old (±9.73) and just over half (53.3%) were male, which was similar to the baseline.

### 3.6 Medication Adherence

The half-reliability coefficient of the medication adherence scale was 0.454.

Of the included 122 patients, eight patients were not evaluated for medication adherence because they did not take any medication, and thus 114 patients were included in this analysis.

### 3.7 Related Factors of Medication Adherence

Univariate analysis found that the main influencing factor of medication adherence was mental health (whether combining with depression, anxiety, bipolar disorder, hallucinations, delusion, cognitive impairments) (*p* < 0.001) and hyposmia (*p* = 0.032).

### 3.8 Changes of Medication Adherence

The adherence score improved at 3-months follow-up (Table 2).

**TABLE 2:** Medication adherence of patients with Parkinson’s disease at baseline and 3-months follow-up.

Items	Baseline (*n* = 114)		3-months follow-up (*n* = 92)		*p*
	Mean ± standard deviation	Median, interquartile range	Mean ± standard deviation	Median, interquartile range	
Adherence	6.19 ± 1.50	6.75 (4.75,7.56)	6.72 ± 1.73	7.00 (6.38,8.00)	0.014

*p* values refer to differences in means.

### 3.9 Frequency of CPCS and Medication Adherence

Frequency of receiving the CPCS was related to improvements in medication adherence, with a statistically significant difference between patients who received the CPCS twice (*p* = 0.011), three or more times (*p* = 0.026), and patients who received CPCS only once.

### 3.10 Association Between CPCS Components and Medication Adherence

Medication adherence was associated with the following components: medication information (*n* = 1, 0.87 points), usage and dosage (*n* = 54, 0.53 points), and medication selection (*n* = 24, 0.16 points). In combination, the following intervention components were also associated with medication adherence: usage and dosage plus medication information (*n* = 1, 1.25 points), medication selection plus medication information (*n* = 1, 1.25 points), medication selection plus usage (*n* = 14, 0.79 points), usage and dosage plus adverse reactions (*n* = 21, 0.73 points), medication selection plus patient education (*n* = 7, 0.64 points), adverse reactions plus patient education (*n* = 9, 0.36 points), usage and dosage plus patient education (*n* = 14, 0.30 points). However, only usage and dosage (*p* = 0.005) reached statistical significance.

### 3.11 Quality of Life (PDQ-39)

The baseline PDQ-39 total scores were 16.26 ± 10.52.

At 3-month follow-up, the number of outpatient visits to receive the CPCS ranged from 1 to 9, with most patients receiving it once (*n* = 60, 49.18%) or twice (*n* = 26, 21.31%). In terms of what was delivered during the CPCS, the most frequently occurring components of the intervention were changes to usage and dosage (57 cases, 36.31%), identification of adverse drug reactions (37 cases, 23.57%), patient education (32 cases, 20.38%), medication selection (24 cases, 15.29%), combined medications/interactions (6 cases, 3.82%), and provision of drug information (1 case, 0.64%).

The 3-months follow-up PDQ-39 total scores was 16.48 ± 10.97 (Table 2).

### 3.12 Factors Related to Quality of Life

After univariate analysis, it was found that the main factor affecting the PDQ-39 total QoL was age (≥70 years) (*p* < 0.001), H&Y stages (*p* < 0.001), constipation (*p* = 0.002), dysphagia (*p* = 0.002), dysuria (*p* = 0.001), “on-off” phenomenon (*p* = 0.003), and “wearing-off” phenomenon (*p* = 0.001). In general, those who were older (≥70 years) and reported dysphagia, constipation, “on-off” phenomenon, “wearing-off” phenomenon, and higher H&Y stage reported poorer QoL.

### 3.13 Changes of Quality of Life

At 3-month follow-up, participants reported improvement in bodily pain subscale (baseline versus 3-months follow-up, 30.04 ± 22.21 versus 23.01 ± 20.98, *p* = 0.037). No statistically significant differences were found in patients’ PDQ-39 total score and mobility, activity of daily living, emotional well-being, stigma, cognitions, and communication subscales when subscales were adjusted (Table 3).

**TABLE 3:** Quality of life of patients with Parkinson’s disease at baseline and 3-months follow-up.

Items	Baseline (*n* = 119)		3-months follow-up (*n* = 92)		*p*
	Mean ± standard deviation	Median, interquartile range	Mean ± standard deviation	Median, interquartile range	
Mobility	21.45 ± 26.24	10.00 (2.50,30.00)	22.74 ± 24.65	15.00 (2.50,35.00)	0.301
Activity of daily living	21.95 ± 22.83	16.67 (4.17,33.33)	23.96 ± 21.70	20.83 (8.33,37.50)	0.188
Emotional well-being	11.13 ± 17.54	4.17 (0.00,16.67)	12.41 ± 19.71	4.17 (0.00,13.54)	0.968
Stigma	12.08 ± 19.84	0.00 (0.00,18.75)	10.87 ± 17.87	0.00 (0.00,18.75)	0.297
Social support	3.22 ± 10.01	0.00 (0.00,0.00)	5.98 ± 12.62	0.00 (0.00,0.00)	0.028
Cognitions	23.16 ± 17.57	25.00 (6.25,31.25)	25.41 ± 17.60	25.00 (12.50,37.50)	0.319
Communication	7.07 ± 13.32	0.00 (0.00,8.33)	7.43 ± 13.89	0.00 (0.00,8.33)	0.827
Bodily pain	30.04 ± 22.21	25.00 (16.67,41.67)	23.01 ± 20.98	16.67 (0.00,41.67)	0.037
PDQ-39 total score	16.26 ± 10.52	14.48 (8.65,21.61)	16.48 ± 10.97	14.56 (8.06,23.10)	0.696

*p* values refer to differences in means.

### 3.14 Frequency of Patients Receiving CPCS and Quality of Life

Frequency of CPCS use was related to activity of daily living subscale (r = 0.208, *p* = 0.047) and the bodily pain subscale (r = 0.232, *p* = 0.026), with statistically significant differences among patients who received the CPCS three or more times and patients who received CPCS for once (*p* = 0.013) or twice (*p* = 0.047). Frequency of receiving the CPCS was not related to PDQ-39 total score (*p* = 0.260), mobility subscale (*p* = 0.539), emotional well-being subscale (*p* = 0.359), stigma subscale (*p* = 0.274), social support subscale (*p* = 0.616), cognitions subscale (*p* = 0.395), and communication subscale (*p* = 0.416).

### 3.15 Association Between CPCS Components and Quality of Life

Total score of PDQ-39 was associated with the following components: patient education (*n* = 32, −0.38 points), adverse reactions (*n* = 37, −0.36 points), usage (*n* = 57, −0.36 points), and medication selection (*n* = 24, −0.16 points). In combination, the following intervention components were also associated with PDQ-39: usage and dosage plus patient education (*n* = 14, −1.94 points), medication selection plus usage (*n* = 14, −1.02 points), medication selection plus patient education (*n* = 7, −0.85 points), usage and dosage plus adverse reactions (*n* = 22, −0.84 points), adverse reactions plus patient education (*n* = 9, −0.80 points). However, only patient education (*p* = 0.005) and usage and dosage combined with patient education (*p* = 0.006) reached statistical significance.

## 4 Discussion

The purpose of this study was to investigate the impact of the CPCS on medication safety and patient reported outcomes. Two databases were used as well as questionnaires consisting of validated measures. For medication safety, the study found that the CPCS could resolve DRPs, improve prescribed medicines, and lower medicines dosages. For patient reported outcomes, the CPCS improved bodily pain subscale and patient adherence, while no significant difference was found in the PDQ-39 total scores and other subscales.

The CPCS was effective in reducing medication risk for PD patients. This is important as movement complications caused by levodopa (such as dyskinesia), which usually occur at about 2–5 years with the medication, could be reduced as a consequence of the CPCS (Turcano et al., 2018; Bressman and Saunders-Pullman, 2019). A randomized double-blind study indicated dosage as a risk factor for motor complications (Olanow and Stocchi, 2018). A Japanese study on data from medical insurance database during 2005–2016 showed the equivalent daily dose of levodopa as 500.0 mg for patients with a PD course of 5–7.5 years (Kasamo et al., 2019). Dosages of levodopa/benserazide in patients receiving CPCS in our hospital was similar to that of these Japanese patients (average daily dose 0.51 ± 0.31 g); compared with patients receiving standard of care, the average dosage was lower, suggesting that the CPCS played a role in adjusting medication dosages and thus reducing the risk of motor complications.

The CPCS as an intervention was intended to improve adherence and QoL (Martinez-Martin et al., 2015). The study found that the CPCS needs to involve frequent contact with patients in order to affect patient outcomes, and highlights a role for pharmacists in following-up service delivery for PD patients. This is not surprising, as previous studies have found that it is important for interventions to be delivered over time and not just on a single occasion to have a sustained effect (Rigotti et al., 2014). As previous studies have found that Chinese patients valued information about their medicines usage and dosage (Yi et al., 2015), during CPCS, pharmacists provided a leaflet of usage and dosage for patients. Our study also found that CPCS components in usage and dosage could improve patients’ medication adherence, which indicated a further strengthened in the future.

Our study also found that patient education or a combination of dosage and patient education can improve patients’ PDQ-39 scores; however, it was not possible to investigate the subscales of this, due to a small sample size in relation to the six intervention components. While this is a limitation of this study, a previous study of PDQ-39 scale in mainland China found that the PDQ-39 scale retesting reliability of the stigma, social support, and cognitive subscales was poor (Luo et al., 2010). Moreover, although some changes in PDQ-39 scores were made and that the scale was validated for Chinese patients, it may not be the most culturally sensitive tool for this population. Other scales for quality of life may be more useful for future studies, such as health-related quality of life (Rajan et al., 2020).

Our study is the first to evaluate the outcomes of CPCS for PD patients using the MRC framework for the development and evaluation of complex interventions (Developing and evaluating, 2019). Prior to undertaking the study reported here we conducted an extensive search of the evidence-base to identify suitable evaluation indicators and outcomes, follow-up time, and intervention components (Yi et al., 2020e2075). As a consequence of this we were able to design a robust investigation incorporating multiple outcome measures, overcoming the limitations of previous studies. We have also been able to establish whether frequency of CPCS delivery has an impact on outcomes, and which intervention components are associated with these, thus providing us with insight into likely intervention mechanisms that can be investigated in more detail in future studies.

Despite this, our study has some limitations. Firstly, not all PD patients could be followed up to capture repeated measures of adherence and PDQ-39 scales, which means that those proving two sets of data may not be representative of all patients. Patients with a higher H&Y stage or combined with non-motor symptoms tended to decline to take part at follow-up, which may have introduced bias in patient selection. Furthermore, the acceptability of and satisfaction with the CPCS was not evaluated in the study, the implications of interventions on DRPs were not recorded. Thirdly, due to limited number of pharmacists providing CPCS, the number of patients who received CPCS is relatively small considering the prevalence of PD in China. Fourthly, although there were no statistically significant differences in age or gender between those patients receiving the CPCS and those who received standard of care, the participants receiving CPCS and standard of care may not be comparable. CPCS was a pharmacist initiated quality improvement campaign and the study was a post-hoc evaluation of its effectiveness, not a study designed in a prospective manner, thus the treatment and measurement were not implemented concurrently for these two groups. Nevertheless, we were not aware of major environmental differences during this period. All study participants were recruited and managed within the single institution.

## 5 Conclusion

A pharmaceutical care intervention designed to support patients with PD can successfully address drug treatment-related problems, improve medication regimens, patients’ QoL, and medication adherence when delivered on two or more occasions. Intervention components consisting of medication dosage adjustments and patient education contributed most to observed intervention outcomes. However, a randomized controlled study is needed to confirm the current findings and explore the mechanisms of interventions.

## Data Availability

The original contributions presented in the study are included in the article/Supplementary Material, and further inquiries can be directed to the corresponding author.
